# Innate Immune Effectors Play Essential Roles in Acute Respiratory Infection Caused by *Klebsiella pneumoniae*

**DOI:** 10.1155/2020/5291714

**Published:** 2020-10-24

**Authors:** Dong Liu, Zhifu Chen, Yue Yuan, Haiming Jing, Jintao Zou, Xiaoli Zhang, Xi Zeng, Weijun Zhang, Quanming Zou, Jinyong Zhang

**Affiliations:** ^1^National Engineering Research Center of Immunological Products, Department of Microbiology and Biochemical Pharmacy, College of Pharmacy, Army Medical University, Chongqing, China; ^2^Department of Clinical Hematology, College of Pharmacy, Army Medical University, Chongqing, China

## Abstract

Innate immune effectors constitute the first line of host defense against pathogens. However, the roles of these effectors are not clearly defined during *Klebsiella pneumoniae* (*K. pneumoniae*) respiratory infection. In the current study, we established an acute pneumonia model of *K. pneumoniae* respiratory infection in mice and confirmed that the injury was most severe 48 h post infection. Flow cytometric assay demonstrated that alveolar macrophages were the predominant cells in BALF before infection, and neutrophils were quickly recruited after infection, and this was in consistent with the kinetics of chemokine expression. Further, we depleted neutrophils, macrophages, and complement pathways *in vivo* and challenged these mice with a sublethal dose of *K. pneumonia*, the result showed that 80%, 60%, and 40% of mice were died in these groups, respectively, while no deaths occurred in the control group. Besides, innate immune effector depleted mice showed higher bacterial burdens in lungs and blood, companied with more severe lung damage and increased levels of cytokine/chemokine expression. These results demonstrated that the innate immune effectors are critical in the early controlling of *K. pneumoniae* infection, and neutrophils are the most important. Thus, alternative strategies targeting these innate immune effectors may be effective in controlling of *K. pneumoniae* respiratory infection.

## 1. Introduction

*Klebsiella pneumoniae* (*K. pneumoniae*) is an opportunistic pathogen that frequently causes life-threatening infections, such as pneumonia, sepsis, and urinary tract infection, in healthy and immune compromised individuals [1]. It accounts for 5-20% of cases of Gram-negative sepsis [2, 3], resulting in significant mortality between 27.4% and 37% [4], and 48% of mortality was observed in patients in ICUs with *K. pneumoniae* infection [5]. Extreme drug-resistant extended-spectrum *β*-lactamases and carbapenemase-producing *K. pneumoniae* isolates have emerged [6–8], and the frequency of the subset of highly invasive and hypervirulent *K. pneumoniae* (hvKp) infections has steadily increased during the past few years [9], making it increasingly difficult to treat *K. pneumoniae* infections with antibiotics. Therefore, alternative strategies for the prevention and treatment of *K. pneumoniae* infections are urgently needed and may indeed be useful. However, the development of these strategies should be based on a deeper understanding of the pathogenic mechanism of *K. pneumoniae* [10].

Innate immune effectors play important roles in controlling local and systemic infections [11–15]. Particularly, neutrophils are quickly recruited to the site of infection and kill the pathogens by oxidative and nonoxidative mechanisms in the early stage of infection [16]. Meanwhile, macrophages are important in both innate and acquired immunity in the respiratory tract and essential in lung defense against infections [17]. Alveolar macrophages are resident mononuclear phagocytes that localize at the boundary between the lungs and external environment, which can recognize and eliminate pathogens before neutrophil recruitment in response to infection [18–22]. The complement system also has the ability to kill invading pathogens by opsonophagocytosis directly [23]. Effective host defense against bacterial infection in the lung mainly depends on the rapid clearance of the pathogens from the respiratory tract, and the failure may result in prolonged infection and bacteria dissemination [24]. However, we know little about the contribution of these innate immune effectors in combating respiratory *K. pneumoniae* infection.

Herein, we established an acute pneumonia model of respiratory *K. pneumoniae* infection in mice and systemically studied the relative contributions of these innate immune effectors during *K. pneumoniae* respiratory infection. Here, we present the protocol on our approach and demonstrate the main findings in this study.

## 2. Materials and Methods

### 2.1. Animals and Ethics Statement

Six- to eight-week-old female BALB/c mice were purchased from Beijing HFK Bioscience Limited Company (Beijing, China) and raised under specific-pathogen-free conditions. Three-month-old female New Zealand white rabbits were purchased from the Animal Center of Chongqing Medical University, housed in cages, and given free access to food and water. All *in vivo* experiments were approved by the Animal Ethical and Experimental Committee of the Army Medical University.

### 2.2. Bacteria and Culture Methods

*K. pneumoniae* reference strain 700721 was purchased from ATCC (Manassas, VA, USA); clinical strains HXT, YYD, and YBQ were provided by the First Hospital Affiliated to Army Medical University (Southwest Hospital). Bacteria were grown on Luria-Bertani (LB) agar plate at 37°C overnight; single colonies were picked and inoculated in LB broth for 3.5 h at 37°C with agitation (220 rpm). To quantify the number of organisms/ml for mice infection, an appropriate dilution of bacteria was made in PBS and determined spectrophotometrically at 600 nm (OD600).

### 2.3. Pneumonia Model

We have established a pneumonia model as described previously [25]. In brief, mice (*n* = 5) were anesthetized with pentobarbital sodium (62.5 mg/kg of body weight) [26] and then challenged with an appropriate dose of different strains of *K. pneumoniae* in a 20 *μ*l volume through noninvasive intratracheal inoculation. Mice were monitored for 7 days for survival rate and body weight. For quantitative bacteriology analysis, blood samples were collected at indicated times from mice eye socket. Then, the mice were euthanized, and the lungs were harvested and homogenized in 1 ml of ice-cold PBS. The blood and homogenate samples were serially diluted in sterile PBS and plated onto LB agar to count the number of CFUs. For histopathology, the lungs of different groups were immediately fixed in 4% paraformaldehyde for 48 h and embedded in paraffin. Sections were cut in 5 *μ*m thick and stained with hematoxylin-eosin. For BALF collection, the sacrificed mice tracheas were exposed and cannulated with a blunt tip needle. The lung lavages were carried out three times with a total of 1.5 ml cold PBS, and the cells in BALF were assayed by flow cytometry.

### 2.4. Complement Susceptibility Assay

Complement susceptibility assay of *K. pneumoniae* was carried out as described previously [27]. In brief, blood samples were collected from a healthy rabbit. For *in vitro* assay, *K. pneumoniae* was centrifuged and suspended in PBS. 50 *μ*l of the bacteria suspension at designated concentration was mixed with 50 *μ*l of rabbit serum in 96-well microplates and incubated at 37°C for 1 h. Serum was either active or heat-inactivated at 56°C for 30 min. Then, the mixture was serially diluted and plated onto LB agar plate to calculate the killing percent.

### 2.5. Depletion of Innate Immune Effectors

Depletion of innate immune effectors was carried out two days before infection. Liposomes containing clodronate, which was a gift of Roche Diagnostics GmbH, were used to deplete alveolar macrophages (AMs) by noninvasive intratracheal route [28]. Each mouse was administrated with 100 *μ*l clodronate-liposomes. Neutrophils were depleted through intraperitoneal injection of cyclophosphamide with a dose of 200 mg/kg [29]. As for the complement pathway, each mouse was intraperitoneally injected with cobra venom factor with a dose of 15 *μ*g, which was able to prevent complement activity for three to six days [30]. Mice (*n* = 5) were challenged with *K. pneumoniae* isolates 48 h later and were sacrificed at the indicated times. Blood and lungs were aseptically collected and used for quantitative bacteriology, histopathology, and inflammatory cytokine/chemokine analysis.

### 2.6. Measurement of Cytokines and Chemokines

IL-1*β*, IL-6, tumor necrosis factor- (TNF-) *α*, C-reactive protein (CRP), MCP-1, MIP-2, and CXCL-1 were reported to be involved in the immunopathogenesis of mouse infection [11, 14, 15, 30]. Levels of these cytokines/chemokines in serum and lung homogenates were determined using ELISA kits (BioLegend, San Diego, CA, USA) according to manufacturer's instructions and reported as pg/ml.

### 2.7. Flow Cytometry Assay

The percentage of AMs and neutrophils in the BALF was determined by flow cytometry as described previously [31]. The BALF cells were blocked using fetal bovine serum for 15 min and then stained with fluorophore-conjugated antibody cocktails for 30 min at 4°C. Surface antibody markers included CD45-BV605 (30-F11), CD11b-PerCp/Cy5.5 (M1/70), CD11c-PE (N418), and Ly6G-FITC (1A8) (BioLegend, San Diego, CA, USA). The AMs in BALF were identified as CD45^+^CD11b^−^CD11c^+^Ly6G^−^ cells while neutrophils were considered as CD45^+^CD11b^+^CD11c^−^Ly6G^+^ cells. All samples were analyzed on a BD FACSCanto™ II flow cytometer (BD Biosciences, Franklin Lakes, NJ, USA).

### 2.8. Statistics

Data were presented as means ± SEM. Survival data for different groups were analyzed using the log-rank test. Bacterial loads and cytokine levels were analyzed using Student's *t* test. Statistics were performed using GraphPad Prism 6.0 (GraphPad Software, Inc., La Jolla, CA, USA) and considered significant when the *P* value was ≤ 0.05.

## 3. Results

### 3.1. Different Strains of *K. pneumoniae* Varied Largely in Virulence

**Figure 1:**
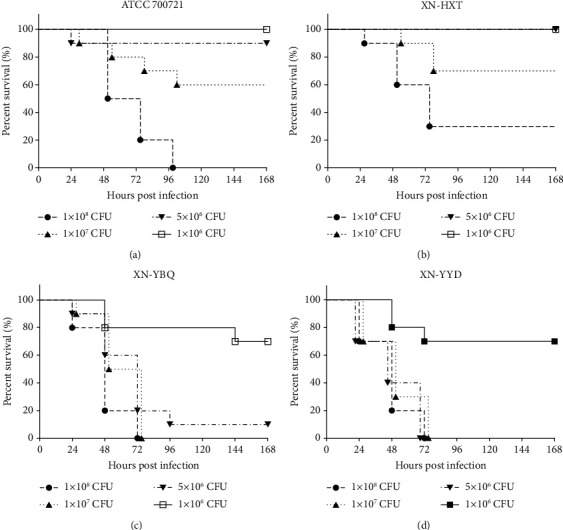
The virulence of different strains of *K. pneumoniae* in mice. Mice (*n* = 10 per group, data were collected from 2 separate experiments) were intratracheally administrated with 1 × 10^6^ CFUs, 5 × 10^6^ CFUs, 1 × 10^7^ CFUs, and 1 × 10^8^ CFUs of *K. pneumoniae* strain YBQ, YYD, HXT, and 700721, respectively. All mice were monitored for 7 days for survival rate calculation.

To investigate the virulence of different strains of *K. pneumoniae*, we selected four isolates from different sources and challenged BALB/c mice intratracheally with different doses (1 × 10^6^, 5 × 10^6^, 1 × 10^7^, and 1 × 10^8^ CFUs) of bacteria. The results showed that most of the deaths occurred in the first three days, suggesting that the infection was more severe during this period. Obviously, 100% of death was observed in mice challenged with 5 × 10^6^ CFUs of strain YBQ and YYD. In contrast, no death was detected in mice challenged with the same dose of strain 700721 and HXT, and 100% mortality occurred in these groups when the CFUs were increased to or even higher than 1 × 10^8^ CFUs (Figure 1). These results illustrated that the virulence of *K. pneumoniae* isolates varied largely from each other, and the virulence of YBQ and YYD was much higher than strain 700721 and HXT. Strain YBQ was chosen as the pathogenic strain in the following studies.

### 3.2. Kinetics of Bacterial Burdens and Histopathology in *K. pneumoniae* Lung Infection

**Figure 2:**
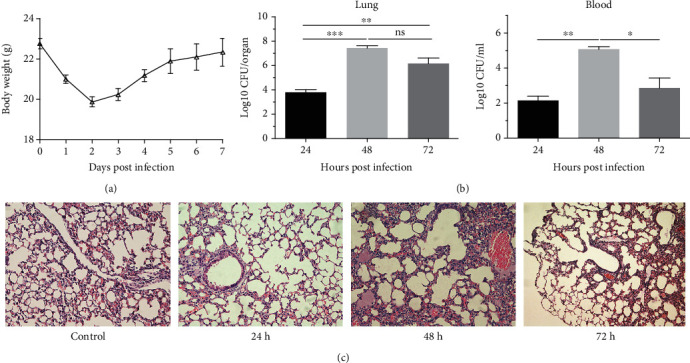
Bacteria burdens and histopathology in pneumonia model of *K. pneumoniae* lung infection. Mice (*n* = 5) were infected intratracheally with 5 × 10^4^ CFUs of strain YBQ, blood, and lung tissue were collected at 24 h, 48 h, and 72 h, respectively. (a) Bodyweight curve of mice was monitored for 7 days. (b) Bacterial burdens in the lungs and blood were counted on Luria-Bertani (LB) plates. (c) Lung sections were stained with hematoxylin-eosin, and histopathology of lung samples was evaluated (magnification = 200×). Statistical analyses were performed by Student's *t* test. *P* < 0.05 (^∗^), *P* < 0.01 (^∗∗^), and *P* < 0.001 (^∗∗∗^) compared to each other.

In order to investigate the kinetics and clearance pattern during YBQ strain lung infection, female BALB/c mice were administrated intratracheally with 5 × 10^4^ CFUs of strain YBQ. Firstly, bodyweight of infected mice was monitored for 7 days; as shown in Figure 2(a), the mice kept losing weight for the first two days after infection and then gained weight since day 3 and gradually recovered from the infection. This result further indicated that the second day was the most severe time point during *K. pneumoniae* lung infection. Next, mice in each group were sacrificed at 24, 48, and 72 hours post infection, respectively, and bacterial burdens in blood and lungs were determined. As shown in Figure 2(b), the highest bacterial burden was observed 48 h after infection and then declined over time. The bacteria slightly disseminated from pulmonary alveolus to the blood 24 h post infection, rapidly increased at 48 h, and then gradually decreased at 72 h.

Next, histological analysis of lung injury was evaluated. As shown in Figure 2(c), the lung histology at 24 h showed mild tissue destruction with inflammatory cell infiltration in the peribronchial areas. 48 h post infection, the lung injury was much more severe with extended alveolar disruption, vascular leakage, bleeding, variable lesions, and inflammatory cell infiltration. The degree of lung injury was alleviated, and the number of inflammatory cells declined in the peribronchial and perivascular areas by 72 h. These results again suggested that the most severe time point of *K. pneumoniae* infection was 48 h after infection.

### 3.3. Profiles of Inflammation during *K. pneumoniae* Lung Infection

**Figure 3:**
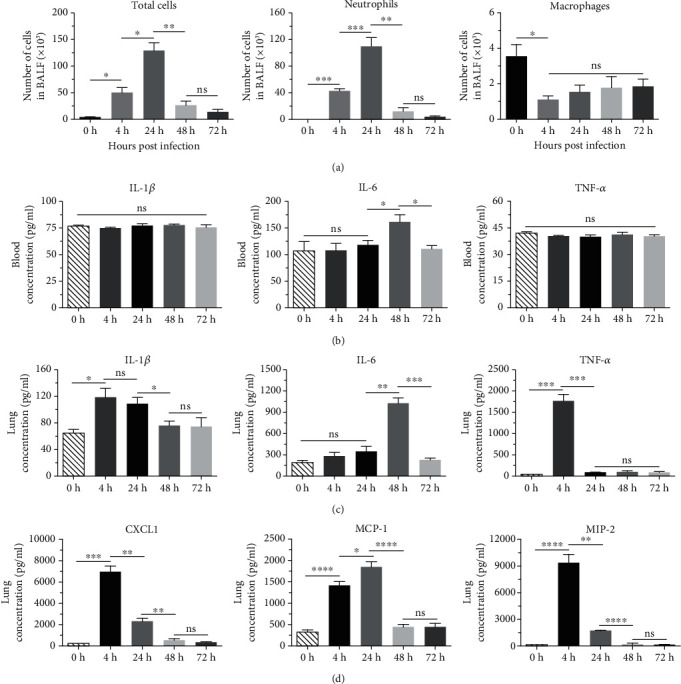
Inflammatory cell infiltration and cytokine/chemokine expression in pneumonia model of *K. pneumoniae* infection. Mice (*n* = 5) were infected intratracheally with 5 × 10^4^ CFUs of strain YBQ, blood, lungs, and bronchoalveolar lavage fluid (BALF) samples were collected at 0 h, 4 h, 24 h, 48 h, and 72 h, respectively. (a) Total cells, macrophages, and neutrophils in BALF samples were quantified through flow cytometry analysis. (b) The levels of IL-1*β*, IL-6, and TNF-*α* in serum and lung samples were detected by ELISA. (c) The levels of MCP-1, MIP-2, and CXCL-1 in lung samples were quantified by ELISA. These assays were performed in triplicate, and representative data from one experiment were expressed as means ± SEM. Statistical analyses were performed by Student's *t* test. *P* < 0.05 (^∗^), *P* < 0.01 (^∗∗^), *P* < 0.001 (^∗∗∗^), and *P* < 0.0001 (^∗∗∗∗^) compared to each other.

Then, we analyzed inflammatory cells in the BALF of infected mice at different time points. The results showed that the number of total cells increased quickly after *K. pneumoniae* infection; a significant difference in the number of total cells was observed 4 h after infection as compared with 0 h, and it reached maximum at 24 h and then decreased rapidly over time (Figure 3(a)). We also determined the subtype of inflammatory cells in BALF; the result showed that the change of neutrophils paralleled the results of total cells, which was approximately 2700-fold as compared to 0 h, and then began to decrease rapidly over time. In contrast, macrophages were the predominant cell type in BALF before *K. pneumoniae* infection, and they were significantly decreased at 4 h and then moderately increased, but no statistic difference was observed during these time points. Obviously, the level of macrophages was significantly lower as compared with neutrophils from 4 h to 72 h. Thus, these results demonstrated that mice infected with *K. pneumoniae* resulted in significant inflammatory cell infiltration, and the change in inflammatory cells was mainly attributed to the infiltration of neutrophils, suggesting that neutrophils played an essential role in the clearance of *K. pneumoniae* lung infection. Since macrophages are the major source of CXCL1 and MIP-2 [32], they may be essential for the recruitment of neutrophils after *K. pneumoniae* lung infection.

Proinflammatory cytokines, such as IL-1*β*, IL-6, and TNF-*α*, are important markers of inflammation [33]. We then determined the levels of these markers in serum and lung samples in infected mice at various time points (4, 24, 48, and 72 h). As shown in Figure 3(b), it was surprising that the levels of IL-1*β* and TNF-*α* in serum showed no difference in different time points as compared with control, whereas the level of IL-6 slightly increased 48 h after infection and reduced to normal level at 72 h, and this was in consistence with the level of CRP (Supplementary Figure 1), which is a marker of inflammation in the body. These results indicated that the inflammation in the blood was limited and controllable after lung infection with a low dose of strain YBQ (5 × 10^4^ CFUs). In contrast, the levels of these cytokines varied largely in lung tissue over time. Both IL-1*β* and TNF-*α* in lung tissues reached a maximum at 4 h. Further, the level of IL-1*β* decreased much slower as compared with TNF-*α*, and the latter quickly decreased to a normal level at 24 h. In contrast, the change of the level of IL-6 in the lungs was similar to that in serum; it reached the highest level at 48 h and then decreased to a normal level. These results demonstrated that proinflammatory cytokines in the lung tissue were more sensitive in response to acute respiratory infection caused by *K. pneumoniae*.

Levels of chemokines are closely related to the recruitment of inflammatory cells [34]. We then measured the levels of CXCL-1, MIP-2, and MCP-1 in lung samples. As shown in Figure 3(c), the level of MCP-1 in lung samples rapidly increased after infection and reached the highest level at 24 h. MIP-2 and CXCL-1 showed a similar tendency in lung samples, both of them reached the highest levels at 4 h and then decreased over time. These results demonstrated that these chemokines responded quickly to *K. pneumoniae* infection, and increased levels were observed 4 h after infection. Further, increased levels of chemokines were observed prior to neutrophils recruitment, which further suggested the importance of neutrophils in the control and clearance of *K. pneumoniae* infection.

### 3.4. Complement Susceptibility Assay

**Figure 4:**
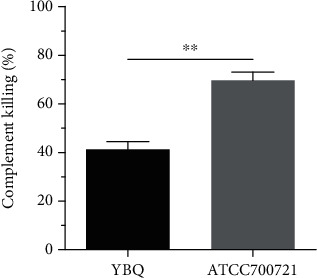
Susceptibility of *K. pneumoniae* to complement-mediated killing. 50 *μ*l of the bacteria suspension (1 × 10^4^ CFUs) was mixed with 50 *μ*l of either active or heat-inactivated rabbit serum in 96-well microplates and incubated at 37°C for 1 h, and viable bacteria were quantified after culture overnight. Complement mediated killing was calculated as follows: (CFU_HI_ − CFU_A_)/CFU_HI_, CFU_HI_ represents heat-inactivated number, and CFU_A_ indicates a complement-active number. This experiment was performed in triplicate, and representative data from one experiment were expressed as means ± SEM. Statistical analyses were performed by Student's *t* test. *P* < 0.01 (^∗∗^) compared to each other.

To determine whether the serum complement-mediated killing played an important role in the clearance of *K. pneumoniae* in blood, we tested the susceptibility of strain YBQ to complement, and the reference strain 700721 was used as a control. Bacteria CFUs were quantified after incubation with active or heat-inactivated serum at 37°C for 1 h. The result showed that YBQ strain exhibited moderate susceptibility with 41% of complement-mediated killing, while strain 700721 showed higher susceptible to complement-mediated killing (73%, Figure 4). This result illustrated that the complement pathway was an important route to clear the bacteria in the blood, which may be essential for alleviating the septicemia, and the virulence of different strains may impact the serum complement susceptibility, which was inconsistent with the result reported previously [35].

### 3.5. Depletion of Innate Immune Effectors Exacerbated *K. pneumoniae* Lung Infection

**Figure 5:**
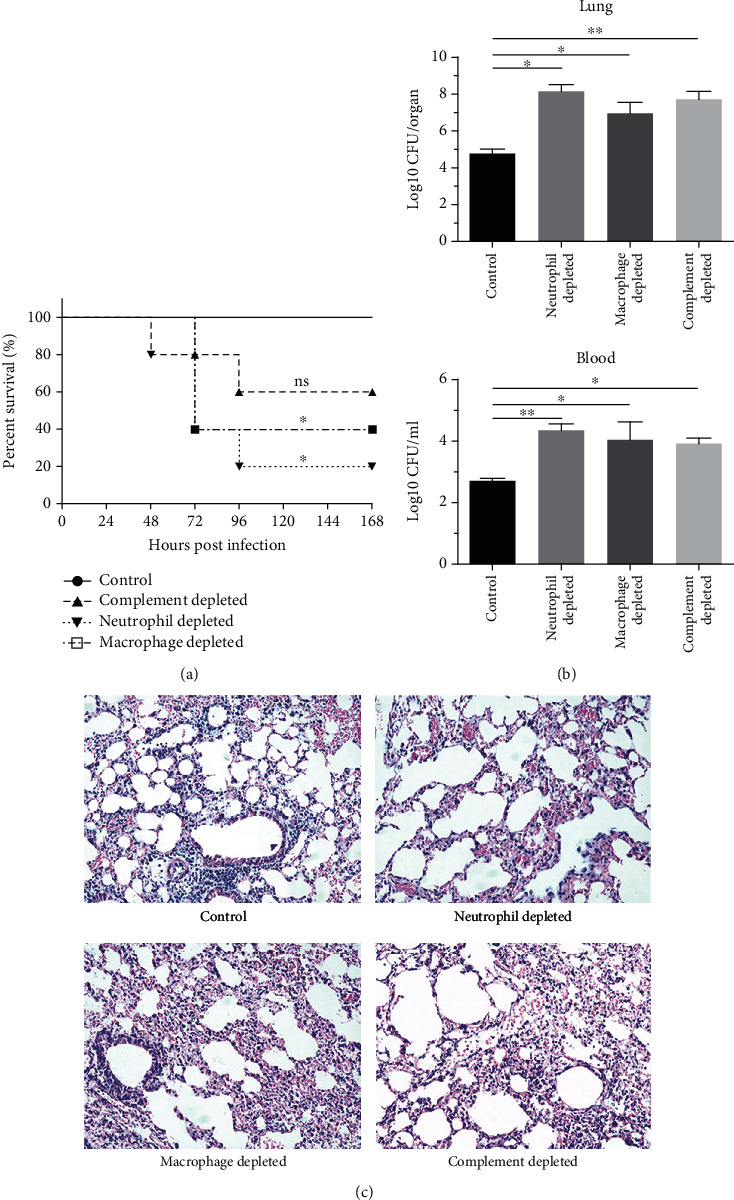
Depletion of innate immune effectors exacerbated *K. pneumoniae* lung infection. Mice (*n* = 5) were infected intratracheally with 1 × 10^6^ CFUs of strain YBQ and blood and lungs were collected 24 h after infection. (a) Survival curves for neutrophil, macrophage, and complement depleted mice were monitored for 7 days. (b) Bacterial burdens in the lungs and blood for different groups were counted on Luria-Bertani (LB) plates. (c) 24 hours after infection, lung tissues in each group were collected, and lung sections were stained with hematoxylin-eosin, and histopathology of different groups was evaluated (magnification = 200×). Statistical analyses were performed by Student's *t* test. *P* < 0.05 (^∗^) and *P* < 0.01 (^∗∗^) compared to control mice.

In order to further evaluate the importance of innate immune effectors in host resistance to *K. pneumoniae* infection, we depleted mice of macrophages, neutrophils, and complement as described above, and flow cytometry assay showed that macrophages and neutrophils were depleted successfully (Supplementary Figures 2 and 3). Two days later, these mice were challenged intratracheally with a sublethal dose of YBQ (1 × 10^6^ CFUs) and monitored for 7 days to calculate the survival rates. As shown in Figure 5(a), no death occurred in the control group, whereas 40% mice died from *K. pneumoniae* infection in the complement depleted group, although there was no statistic difference. Further, 80% and 60% of mice died in the neutrophil and macrophage depleted group, respectively, which were significantly higher than the control group. These results indicated that all the three innate immune effectors played important roles in the control of the infection, and among which neutrophils were the most important.

For bacterial burdens and histopathology analysis, mice were sacrificed 24 h after challenge, and blood and lungs were collected, respectively. The results showed that the bacterial burdens in the lungs for neutrophils, macrophages, and complement depleted mice were 2400-fold, 150-fold, and 860-fold higher as compared with the control group. Meanwhile, the bacterial burdens in blood were inconsistent with that in the lungs, which were 45-fold, 21-fold, and 16-fold higher as compared with the control group (Figure 5(b)). The lung histology for different groups also showed different degrees of tissue destruction. As shown in Figure 5(c), lungs of innate immune effector depleted mice appeared more severe with extended variable lesions and accumulated extensive inflammatory cells, especially for neutrophil and macrophage depleted mice.

### 3.6. Inflammatory Profiles in Innate Immune Effector Depleted Mice during *K. pneumoniae* Lung Infection

**Figure 6:**
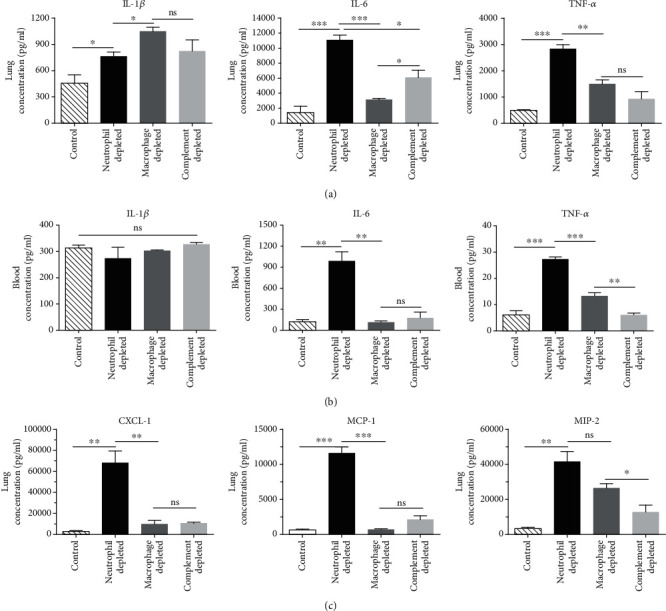
Depletion of innate immune effectors resulted in increased levels of cytokines/chemokines in *K. pneumoniae* lung infection. (a) The levels of IL-1*β*, IL-6, and TNF-*α* in the lung were detected by ELISA. (b) The levels of IL-1*β*, IL-6, and TNF-*α* in blood samples were detected by ELISA. (c) The levels of MCP-1, MIP-2, and CXCL-1 in lung samples were measured by ELISA. These assays were performed in triplicate, and representative data from one experiment were expressed as means ± SEM. Statistical analyses were performed by Student's *t* test. *P* < 0.05 (^∗^), *P* < 0.01 (^∗∗^), and *P* < 0.001 (^∗∗∗^) compared to each other.

The levels of IL-1*β*, IL-6, and TNF-*α* in serum and lung samples were compared between innate immune effector depleted mice and control mice 24 h after infection (Figures 6(a) and 6(b)). In lung samples, the levels of all the three cytokines were significantly increased in neutrophil depleted mice. In contrast, only the level of IL-1*β* was increased in macrophage depleted mice, and the levels of IL-1*β* and IL-6 were slightly increased in complement depleted mice. Meanwhile, the highest levels of IL-6 and TNF-*α* were observed in neutrophil depleted mice, whereas a higher level of IL-1*β* was detected in macrophage depleted mice compared with neutrophil depleted mice. The levels of IL-6 and TNF-*α* in blood in each group were inconsistent with that in lung samples, whereas the level of IL-1*β* in blood showed no difference between innate immune effector depleted mice and control mice.

The levels of MCP-1, MIP-2, and CXCL-1 in lung samples were also measured (Figure 6(c)). The results showed that the highest levels of all the three chemokines were observed in neutrophil depleted mice, which were 18-fold, 25-fold, and 12-fold higher as compared with that in control mice, this result again confirmed that neutrophils played an essential role in controlling of *K. pneumoniae* infection. Meanwhile, the level of MIP-2 was 8-fold and 4-fold higher in macrophage and complement depleted mice as compared with that in the control group. Together, these results indicated that all three innate immune effectors played important roles in the clearance of YBQ strain infection, and the neutrophils were more important compared to macrophages and complement.

### 3.7. Innate Immune Effectors Exhibited Synergy Effect in *K. pneumoniae* Lung Infection

**Figure 7:**
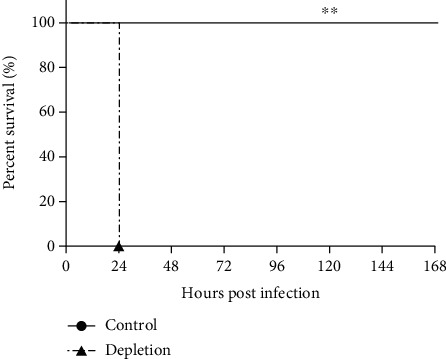
Innate immune effectors exhibited synergy effect in *K. pneumoniae* lung infection. Three pathways were simultaneously disrupted, and mice (*n* = 5) were intratracheally administrated with 5 × 10^5^ CFUs of strain YBQ 48 h later. The mice were monitored for 7 days to calculate the survival rate. Statistical analyses were analyzed using the log-rank test. *P* < 0.01 (^∗∗^) compared to control mice.

In order to further demonstrate our conclusion, we depleted the three innate immune effectors simultaneously to investigate the importance of these effectors in controlling of *K. pneumoniae* infection. As shown in Figure 7, when challenged with a lower dose of strain YBQ (5 × 10^5^ CFUs), all mice depleted these innate immune effectors died within 24 h post infection, while control mice that were not depleted of these effectors showed no death throughout the monitor period. In contrast, survived mice were observed when depleted one of these three effectors and challenged with a higher dose of YBQ strain (1 × 10^6^ CFUs). This result illustrated that these three innate immune effectors resulted in increased susceptibility and exhibited a synergy effect in controlling of *K. pneumoniae* lung infection.

## 4. Discussion

The fatal infections caused by *K. pneumoniae* and difficult-to-treat properties highlighted the importance to develop new strategies to prevent or treat related infections. In this study, we established a pneumonia model through noninvasive intratracheal infection in mice and found that neutrophils, macrophages, and complement were important in host defense against *K. pneumoniae* infection. Thus, alternative strategies could focus on enhancing the response of these innate immune effectors to prevent or cure the infection. For example, there is a report that miR-223 was able to regulate the bactericidal capacity of neutrophils at wound sites [36], and thus targeting miR-223 might be of therapeutic benefit for preventing *K. pneumoniae* infection.

In this study, four different strains of *K. pneumoniae* were used to determine the virulence. The results showed that these strains exhibited significant differences in virulence in the pneumonia model, and we found that the virulence of strain YBQ and YYD was stronger than strain 700721 and HXT. String test of strain YBQ and YYD showed greater than 5 mm “string” between an inoculating loop and a plated bacteria colony [37], which indicated that both of them exist as hypermucoviscous phenotype, and this phenotype is associated with increased expression of extracellular polysaccharides, and the latter is a major virulence factor of *K. pneumoniae* [9].

MCP-1, MIP-2, and CXCL-1 are important chemokines involved in the recruitment of inflammatory cells. MIP-2 and CXCL-1 are mainly secreted by macrophages and regulates the migration of neutrophils to inflammation sites [38], whereas MCP-1 exhibits chemotactic activity of monocytes and mediates macrophage function [39, 40]. Our results showed that the levels of CXCL1 and MIP-2 quickly reached peak 4 h after infection, and these chemokines may be secreted by macrophages because they were the majority cells in BALF before infection. Obviously, increased levels of these chemokines resulted in rapid recruitment of neutrophils to the site of infection, which became the dominant cells in BALF 24 h after infection. MCP-1 was involved in the recruitment of macrophages and it reached a maximum level 24 h after infection; as we can see from our result, the number of macrophages slightly increased after 24 h although there was no statistical difference. Our results also showed that the level of chemokines in innate immune effector depleted mice was much higher as compared with control mice, which may be attributed to the fact that the host should express higher levels of chemokines to recruit inflammatory cells to the site of infection, although these cells were depleted.

The importance of neutrophils, macrophages, and complement in preventing *K. pneumoniae* lung infection was further confirmed by depletion of these components *in vivo*, which resulted in increased mortality, bacterial burdens, more severe lung damage, and enhanced levels of cytokine/chemokine expression when compared to control mice. Cyclophosphamide-mediated depletion of neutrophils obviously sensitized mice to intratracheal infection, allowed for extrapulmonary dissemination, and led to as high as 80% of mortality. Compared with neutrophils, the importance of macrophages or complement was slightly weaker, but still essential. Our results showed that a combined depletion of these host defense effectors exacerbated the infection, which was confirmed by the fact that all the mice in this group died even with a decreased challenge dose, suggesting that these defense effectors worked in synergy to combat *K. pneumoniae* lung infection.

## 5. Conclusions

Collectively, we established a pneumonia model through noninvasive intratracheal infection and investigated the kinetics and clearance of *K. pneumoniae* infection in this study. Further studies demonstrated that host innate defense elements played essential roles in early control of *K. pneumoniae* lung infection, and neutrophils were the key effectors for improved survival, thus, alternative strategies may focus on these innate immune effectors to improve the control of *K. pneumoniae* infection.

## Data Availability

The authors confirm that the data supporting the findings of this study are available within the article and its supplementary materials.

## Supplementary Materials

Supplementary Materials Supplementary Figure 1: C-reactive protein (CRP) in serum of mice after *K. pneumoniae* strain YBQ lung infection. Mice (*n* = 5) were infected intratracheally with 5 × 10^4^ CFUs of strain YBQ blood were collected at 0 h, 24 h, and 48 h, respectively. The level of CRP in blood samples was measured using an ELISA kit. Data were presented as means ± SEM. Statistical analyses were performed by Student's *t* test. Supplementary Figure 2: efficacy of alveolar macrophage (AMs) depletion. Mice (*n* = 5) were intratracheally administrated with phosphate-buffered saline (PBS) or clodronate-liposomes; bronchoalveolar lavage fluid (BALF) samples were collected at day 0, day 2, day 3, and day 4, respectively, and then analyzed using flow cytometry. Blocked BALF samples were stained with CD45, CD11b, and F4/80. Gates representing AMs were illustrated, and the event frequencies were indicated. Supplementary Figure 3: efficacy of neutrophil depletion. Mice (*n* = 5) were intraperitoneally injected with phosphate-buffered saline (PBS) or cyclophosphamide, and bronchoalveolar lavage fluid (BALF) samples were collected at 4 h and 24 h after infection with 5×10^4^ CFUs of YBQ, respectively, and then analyzed using flow cytometry. Blocked BALF samples were stained with CD45, CD11b, CD11c, and Ly-6G. Gates representing neutrophils were illustrated, and the event frequencies were indicated.
