# Supplementary figures and images for: Innate Immune Effectors Play Essential Roles in Acute Respiratory Infection Caused by Klebsiella pneumoniae
Source: J Immunol Res. 2020 Oct 24;2020:5291714. doi: 10.1155/2020/5291714 (PMC7607282; doi:10.1155/2020/5291714)

Supplementary Figure 1


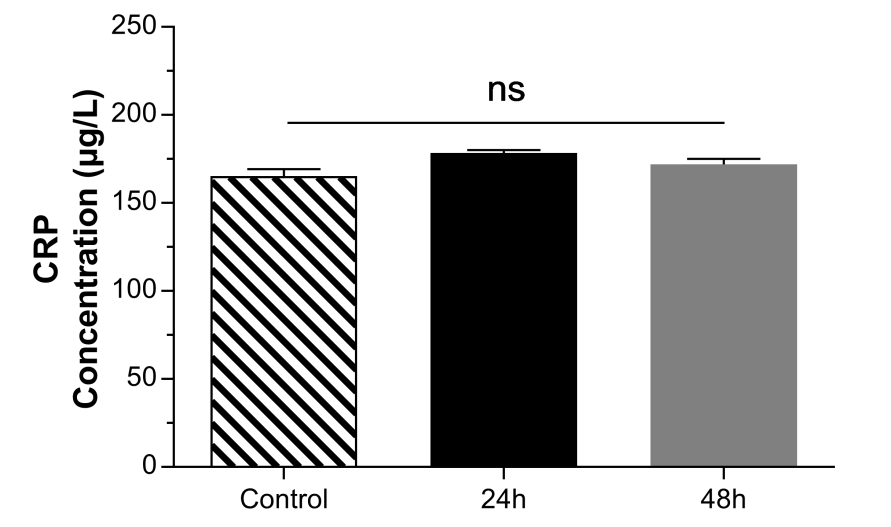


Supplementary Figure 2
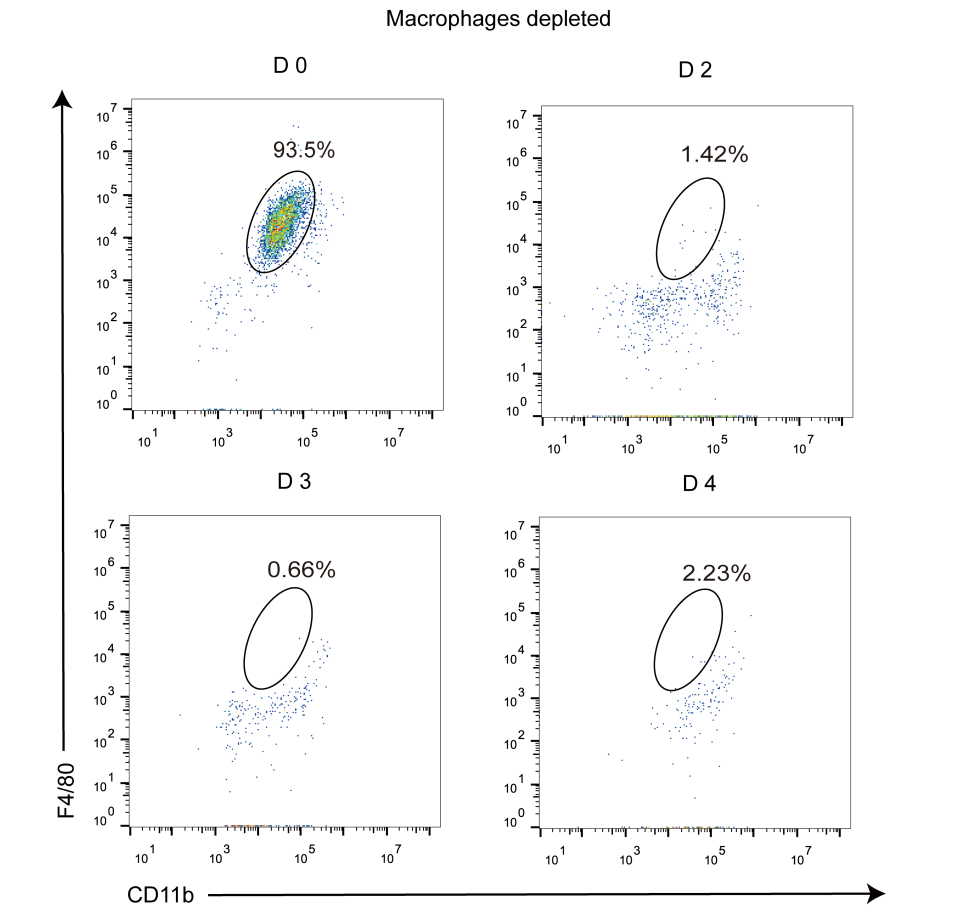


Supplementary Figure 3


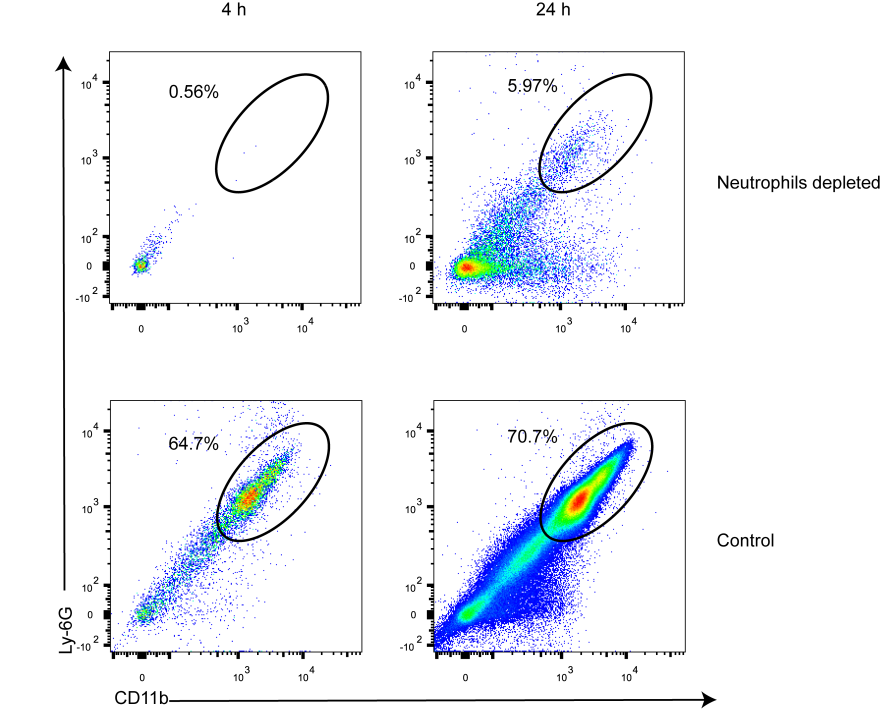

Supplement: Supplementary Materials — Supplementary Figure 1: C-reactive protein (CRP) in serum of mice after K. pneumoniae strain YBQ lung infection. Mice (n = 5) were infected intratracheally with 5 × 10^4^ CFUs of strain YBQ blood were collected at 0 h, 24 h, and 48 h, respectively. The level of CRP in blood samples was measured using an ELISA kit. Data were presented as means ± SEM. Statistical analyses were performed by Student's t test. Supplementary Figure 2: efficacy of alveolar macrophage (AMs) depletion. Mice (n = 5) were intratracheally administrated with phosphate-buffered saline (PBS) or clodronate-liposomes; bronchoalveolar lavage fluid (BALF) samples were collected at day 0, day 2, day 3, and day 4, respectively, and then analyzed using flow cytometry. Blocked BALF samples were stained with CD45, CD11b, and F4/80. Gates representing AMs were illustrated, and the event frequencies were indicated. Supplementary Figure 3: efficacy of neutrophil depletion. Mice (n = 5) were intraperitoneally injected with phosphate-buffered saline (PBS) or cyclophosphamide, and bronchoalveolar lavage fluid (BALF) samples were collected at 4 h and 24 h after infection with 5×10^4^ CFUs of YBQ, respectively, and then analyzed using flow cytometry. Blocked BALF samples were stained with CD45, CD11b, CD11c, and Ly-6G. Gates representing neutrophils were illustrated, and the event frequencies were indicated. [file 5291714.f1.docx]
